# Assessment of malignant melanoma lesions using violet‐light dermoscopy: A case report

**DOI:** 10.1111/1346-8138.16389

**Published:** 2022-04-17

**Authors:** Juri Shu, Yosuke Yamamoto, Kazuhiro Aoyama, Yaei Togawa, Takashi Kishimoto, Hiroyuki Matsue

**Affiliations:** ^1^ Department of Dermatology Chiba University Graduate School of Medicine Chiba Japan; ^2^ Department of Molecular Pathology Chiba University Graduate School of Medicine Chiba Japan

**Keywords:** melanin, melanoma, near‐ultraviolet, resection margin, violet‐light dermoscopy

## Abstract

Malignant melanomas often present with irregular shapes and in multiple shades of brown under white light. Dermoscopy is used to diagnose malignant melanomas; nevertheless, it is often difficult to differentiate malignant melanoma from healthy pigmented skin. The DZ‐D100 dermoscope (Casio Computer) is a digital camera equipped with a white light‐emitting diode (LED) and a violet LED, which can capture non‐polarized/polarized conventional dermoscopy images (CDS) as well as violet‐light dermoscopy (VLD) images. Since the absorption wavelength of melanin approaches that of ultraviolet rays, VLD with a wavelength of 405 nm can be used to visualize it. This camera allows three images with the same composition to be captured simultaneously. In this case, we performed dermoscopy with DZ‐D100 to determine the surgical resection margins of a melanoma of the heel in a 76‐year‐old woman. The pale‐colored lesions that were difficult to demarcate by CDS were clearly visible by VLD, presenting as dark areas in the grayscale images. Preoperatively determined lesion boundaries with CDS in combination with VLD were histologically more accurate than those with conventional CDS alone. Therefore, the combination of CDS and VLD may reveal the distribution of subtle pigmentation of fine melanin in the skin, making it easier to distinguish between lesions and healthy skin. As one of the limitations, parts of the heel with thick stratum corneum were also observed to be dark gray in the VLD images. Therefore, the evaluation of pigment lesion should be performed by comparing both CDS and VLD.

## 1 INTRODUCTION

**FIGURE 1:**
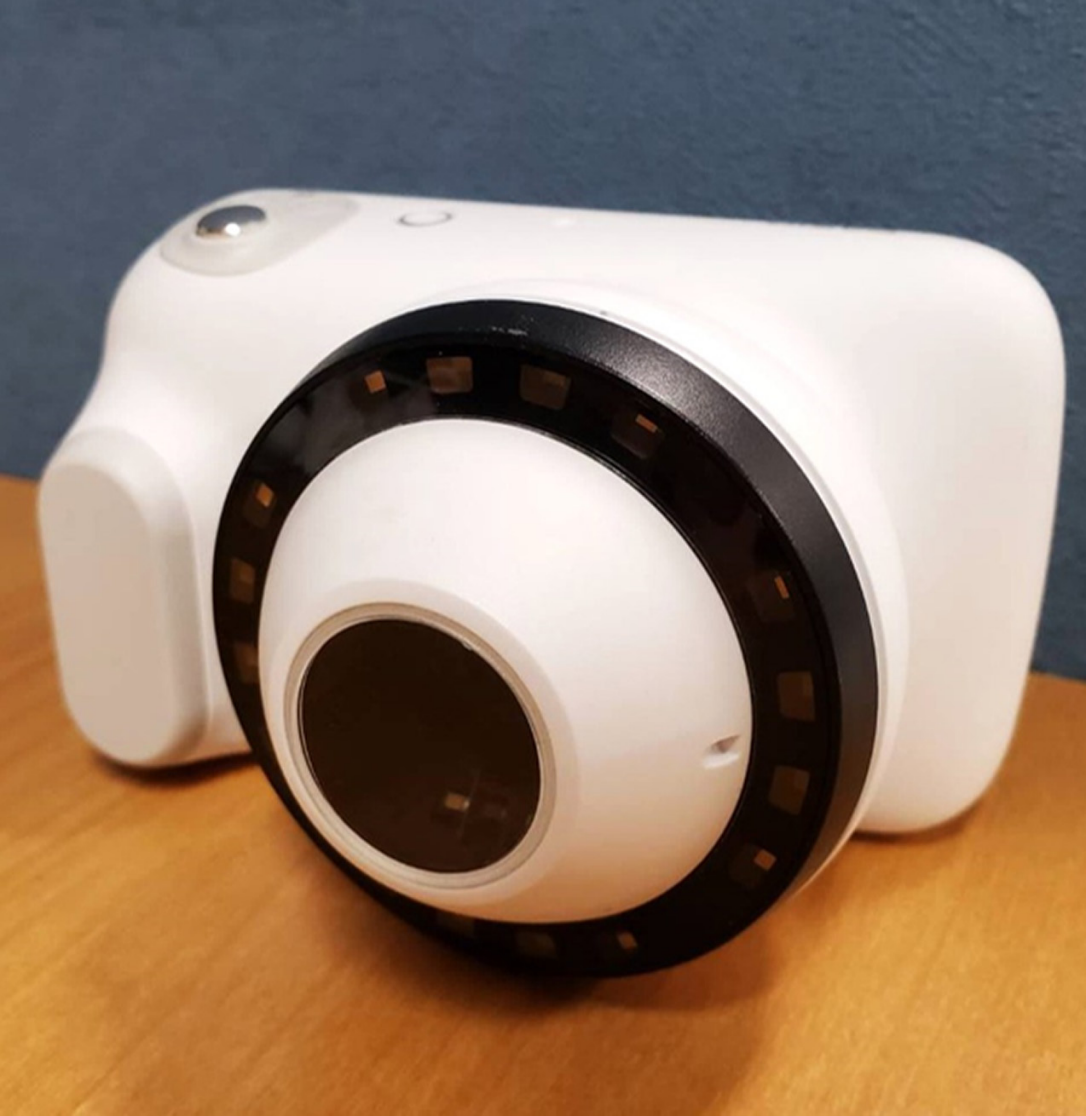
The DZ‐D100 (Casio Computer). This is a digital camera equipped with a violet light‐emitting diode (LED) in addition to the usual white light LED, which can capture non‐polarized conventional dermoscopy images (CDS), polarized dermoscopy images, and violet‐light dermoscopy (VLD) images with a single shutter click, in addition to clinical photographs

Malignant melanomas often present with irregular shapes and in multiple shades of brown under white light.^1^ In pale‐toned melanoma, it is often difficult to distinguish the boundary between the lesion and adjacent healthy skin with the naked eye, even with conventional dermoscopy (CDS). The DZ‐D100 dermoscope (Casio Computer) is equipped with a violet light‐emitting diode (LED), which is a near‐ultraviolet (UV) light with a wavelength of 405 nm, in addition to a conventional white LED (Figure 1). Since the absorption wavelength of melanin contains violet light, the combination of CDS and violet‐light dermoscopy (VLD) may reveal the distribution of subtle pigmentation of fine melanin in the skin, making it easier to distinguish between lesions and healthy skin.

We herein present a case of acral melanoma that was photographed by CDS and VLD before surgery to demarcate lesions and determine resection margins, with the consent of the patient.

## 2 CASE REPORT

**FIGURE 2:**
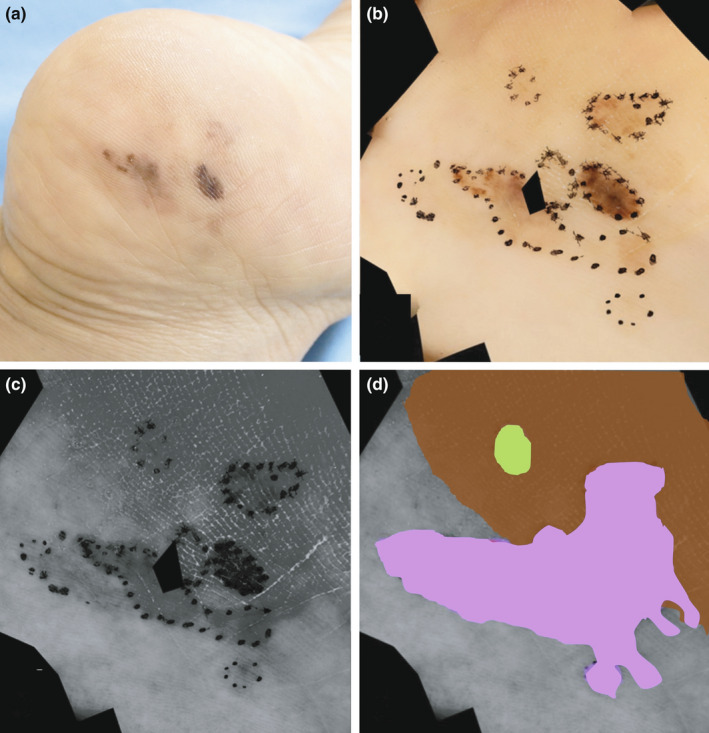
Malignant melanoma of the heel. (a) Clinical photograph of the lesion showing a 30 mm × 25 mm‐sized pigmented macule on the lateral aspect of the left heel. (b) Conventional dermoscopy images (CDS) that combines multiple images. For this reason, the center of the image is missing, and the edges are jagged. Based on the clinical photographs and CDS, the area that appeared to be the lesion was marked with a black dotted line. (c) Violet‐light dermoscopy (VLD) image (similar to Figure 2b, the center of the image is missing): dark gray areas extended beyond the area indicated by the black dotted lines in (b). (d) The area of melanoma identified by the combined use of CDS and VLD is displayed in light purple pseudocolor. In contrast, the region shown in light green was obscured in the VLD image, although it was observed as melanoma lesions on CDS images. The region where the stratum corneum of the heel was thick that appeared grayish black in the VLD image is shown in brown

A 76‐year‐old woman presented to our hospital with pigmented macules on her left heel. She noticed the lesions 10 years earlier but did not seek medical attention until her family advised her to see a physician. The lesions were distinct black‐brown hyperpigmented macules (~30 mm × 25 mm) on the lateral aspect of the left heel (Figure 2a). Using CDS, a parallel ridge pattern was observed inside the irregular and scattered, light brown structureless area, but the boundary with normal skin was unclear.

**FIGURE 3:**
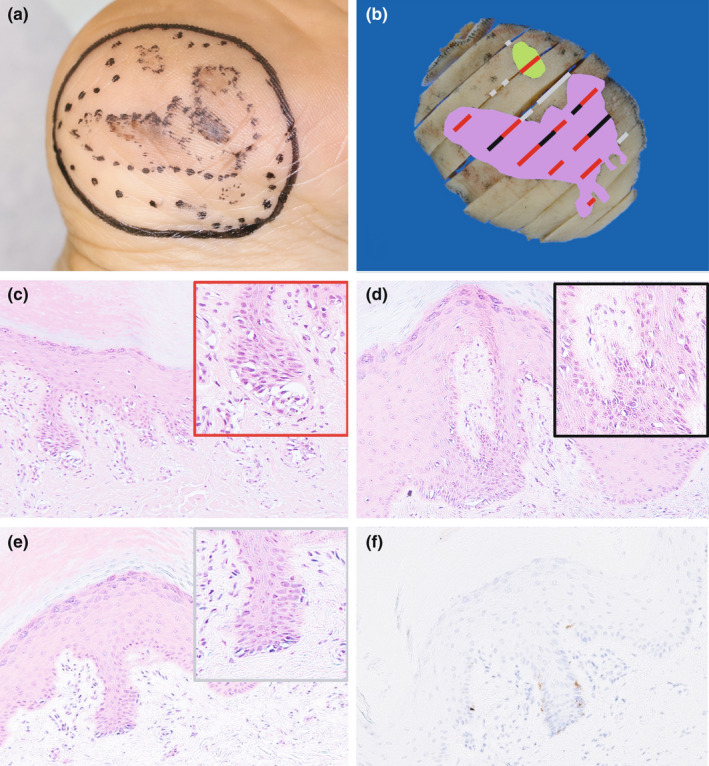
Comparison between tissue section and the pathological lesion. (a) The actual resection line was plotted (solid black line) with 5‐mm margin from the proposed melanoma boundary. (b) Correspondence between excised specimen and histological site of melanoma (each strip‐shaped cut surface was sampled). The areas of suspected melanoma, observed preoperatively using both conventional dermoscopy (CDS) and violet‐light dermoscopy (VLD), were confirmed histologically to be melanoma. The areas identified by CDS alone are indicated by a red line, and the areas determined by adding VLD are indicated by a black line. Some areas of melanoma were missed using both CDS and VLD (shown by the gray line). (c–e) Representative histopathology corresponding to each line. (c) The lesions confirmed by CDS (hematoxylin–eosin staining; 100×); the red frame shows the highly magnified image (200×). Atypical melanocytes with clear cytoplasm proliferate within the rete ridges, forming small tumor nests in some areas. (d) The lesions identified by adding VLD to CDS (hematoxylin–eosin staining; 100×); the black frame shows the highly magnified image (200×). The proliferation of atypical melanocytes is observed in the rete ridges, but the distribution is sparser than in the lesion confirmed by CDS. (e) A section of a lesion that could not be identified by the combined use of CDS and VLD (hematoxylin–eosin staining; 100×); the gray frame shows the highly magnified image (200×). The proliferation of atypical melanocytes is almost invisible. (f) Melan‐A staining of the same slice as in Figure 3e; The distribution of melanin is sparse (100x)

We made a provisional diagnosis of melanoma and then evaluated the dermoscopic images acquired by CDS plus VLD to demarcate the lesions and determine resection margins. Observation with CDS alone (Figure 2b) showed irregular brown spots, and we marked the suspected lesion areas with a black dotted line and compared these areas with clinical photographs. Additionally, examination of VLD images revealed very pale pigmented areas that were difficult to identify as part of the lesion on CDS. In grayscale images obtained under violet light, they appeared as black‐gray areas extending beyond the black dotted lines (Figure 2c). However, as shown in Figure 2d, the healthy part of the heel with a thick stratum corneum (brown area in CDS) also showed a black‐gray hue in VLD (Figure 2c), and the boundary between the lesion and healthy skin was unclear. Therefore, the gray‐black region of the VLD image, which was continuous from the region considered to be melanoma on the CDS image, was determined to represent the true boundary of the lesion (light purple pseudocolored area). Contrastingly, one area (light green) was obscured on VLD images although it was clearly observed as a melanoma area on CDS images. Consequently, both CDS and VLD images were used complementarily to determine the extent of the lesion and to plot the preoperative resection area. Total excision was performed in one piece with a 5‐mm margin from the suspected melanoma lesion (Figure 3a).

Tissue specimens were prepared from the excised tissue at specific locations. The lines drawn on the CDS image in Figure 3b indicate the histologically confirmed areas of melanoma cells. The red and black lines correspond to the areas where melanoma was suspected in CDS and VLD before surgery, and the gray lines correspond to the areas where melanoma was not suspected (Figure 3b). In the area of the melanoma identified only by CDS (Figure 3c), atypical melanocytes with clear cytoplasm and atypical nuclei were observed. In contrast, the area of melanoma that could be identified by supplementing CDS with VLD showed somewhat sparse proliferation and atypical melanocytes (Figure 3d). Consequently, in the areas of the melanoma that could not be identified by the combined use of CDS and VLD, the distribution of atypical melanocytes was sparse on hematoxylin–eosin (Figure 3e) and melanin‐A (Figure 3f) staining. The lesion was *in situ* and the margins were negative. The patient is alive without recurrence or metastatic disease for >2 years after surgical treatment.

## 3 DISCUSSION

Ultraviolet cameras are commonly used in the field of cosmetology because they can clearly detect lightly pigmented spots on the skin, observed as gray‐black areas in the grayscale image, that cannot be seen with a typical color camera.^2^ Additionally, UV cameras are occasionally used to evaluate skin diseases associated with pigment abnormalities such as pigment regeneration in vitiligo vulgaris.^3^, ^4^, ^5^ Although these cameras use light sources in the UVAI wavelength range of 340–380 nm, violet light (380–430 nm), which is visible, can also identify faint melanin spots that are not visible to the naked eye.^2^ The DZ‐D100 dermoscope is a new digital camera supplied with a violet LED (405 nm) in addition to the common white light LED. Recently, Sano *et al*.^6^ reported a similar case in which the border of melanoma was clarified using DZ‐D100 dermoscopy with VLD, but there was no description of histopathological examination of the lesion.

In our case, we evaluated the VLD and CDS images together to clarify the areas of the lightly pigmented skin and histologically confirmed that area was melanoma. However, as a limitation of this technique, we found it difficult to distinguish lesions using VLD in areas where the histological distribution of melanin and melanocytes is sparse. Additionally, UV cameras cannot quantify the difference between black and brown stains, making it difficult to tell the difference in color.^2^ Furthermore, hyperpigmented areas observed as dark gray discoloration using VLD can also occur in other diseases and secondary melanin deposition. Furthermore, in our experience, it is expected that dark gray tones will be observed in VLD due to erythema (hemoglobin), in heels with thick stratum corneum, and shadows associated with irregularities on the skin surface (data not shown). In our case, we determined that only the dark gray area in the VLD image adjacent to the lesion, confirmed by CDS, was melanoma. In regions of the VLD image that were darker due to the thick stratum corneum of the heel (Figure 2d), it was difficult to distinguish the boundary of the melanoma by VLD alone. As Lee *et al*.^5^ proposed, we carefully evaluated the pigmented regions by combining UV photography (VLD) with visible‐light photography (CDS) to improve diagnostic accuracy.

In conclusion, the combination of VLD and CDS may be able to demarcate very thin lesions of melanoma that are difficult to see with the naked eye and facilitate differentiation between lesions and healthy skin. However, if the distribution of melanin is very sparse or if the lesion is located on a heel with a thick stratum corneum, it may be difficult to visually recognize the lesion. Therefore, it is helpful to evaluate the images captured by both CDS and VLD carefully to demarcate lesions.

## CONFLICT OF INTEREST

The Department of Dermatology at Chiba University received payment from Casio Computer, for providing dermoscopy images and annotation for their website (https://dzimage.casio.jp). Dr. Togawa has received payment from Casio Computer for speaking on their behalf. Drs. Shu, Yamamoto, Aoyama, Kishimoto, and Matsue have no conflicts of interest to declare.
